# Surgical treatment of elderly patients with infrarenal abdominal aortic aneurysm

**DOI:** 10.1186/1471-2318-11-S1-A6

**Published:** 2011-08-24

**Authors:** E Cappello, F Spinetti, M Di Lorenzo, E Franco

**Affiliations:** 1 Unit of Vascular Surgery Rummo Hospital, Benevento, Italy

## Background

The traditional surgical treatment for abdominal aortic aneurysm (AAA) is now well codified in vascular surgery with a perioperative mortality rate that has gradually declining in recent years. The introduction of endovascular techniques has led the indications for surgery to be reviewed. The results of surgery in patients over eighty are fairly well defined: it is encoded that age affects only a small part of the immediate results in terms of overall mortality, while it is a significant factor in increasing rates of perioperative major morbidity, in particular cardiac and respiratory diseases. The purpose of this article is to assess the octogenarian patients who are not candidates for treatment with endoprosthesis.

## Materials and methods

The characteristics of the proximal neck, distal and size influence the feasibility of enablers and results of endovascular treatment. All patients who do not respect these features are handled by us with traditional surgery. The high prevalence of ischemic heart disease in patients with AAA is a major cause of morbidity and mortality (46.2% of early mortality, late mortality of 17.7%) in the surgical treatment of AAA (Won et al. Validation of selective cardiac evaluation prior to aortic aneurysm repair).

Literature data show that if coronary angiography is performed routinely in patients with AAA awaiting surgery, the frequency of hemodynamically significant stenosis varies from 46 to 75%. (Utoh et al.) For the reasons that all patients, twice in our experience can not be treated by endovascular undergo coronary angiography before surgery for AAA.

## Results

In our series, since 2002, we have operated on 560 patients of which 282 are of AAA ultra octogenarians.

Of these, 224 have been processed with endoprosthesis and 58 to surgical repair of aortic graft. 100% of those operated with traditional surgery had a short proximal neck, in 70% with the involvement of a renal artery aneurysm. 42% of all patients had a coronary heart disease that was treated preoperatively with coronary stents. Among the octogenarians the operative mortality of patients undergoing surgical repair for AAA was 4%.

## Conclusions

The advent of endoprotesis has certainly improved the survival rate and morbidity of elderly patients with risk of AAA rupture. A selection of the patients, careful study of cardiac risk and treatment of coronary artery disease and carotid artery before surgery are prerequisites to reduce perioperative complications.
